# Preparing Medical Teachers for Small-Group Active Learning: A Design-based Research Study

**DOI:** 10.5334/pme.1759

**Published:** 2025-09-18

**Authors:** Jan Willem Grijpma, Anne de la Croix, Diana H. J. M. Dolmans, Martijn Meeter, Robbie H. J. M. Grooten, Rashmi A. Kusurkar

**Affiliations:** 1 Amsterdam UMC location Vrije Universiteit Amsterdam, Research in Education, De Boelelaan 1118, Amsterdam, The Netherlands; 2 Centre for Teaching & Learning, Vrije Universiteit Amsterdam, Amsterdam, The Netherlands; 3 LEARN! Research Institute for Learning and Education, Faculty of Psychology and Education, Vrije Universiteit Amsterdam, Amsterdam, The Netherlands; 4 Department of Educational Development & Research, School of Health Professions Education, Faculty of Health, Medicine and Life Sciences, Maastricht University, Maastricht, The Netherlands; 5 Amsterdam UMC location Vrije Universiteit Amsterdam, Faculty of Medicine, Amsterdam, The Netherlands; 6 Amsterdam Public health, Quality of Care, Amsterdam, The Netherlands

## Abstract

**Introduction:**

Small-group active learning methods can enhance student learning, but engaging students in these methods can be challenging for teachers. Therefore, Faculty Development Initiatives (FDIs) typically focus on medical teachers’ proficiency in active learning strategies, yet transferring these strategies to actual teaching practices remains problematic. To address this, we designed, implemented, and evaluated an FDI aimed at stimulating this transfer to support medical teachers in facilitating active learning.

**Methods:**

We conducted a Design-Based Research study with 34 new medical teachers in a small-group active learning course. The FDI combined Self-Directed Learning with on-the-job and off-the-job learning. Surveys and interviews were used in two separate iterations of the FDI to evaluate how transfer was stimulated. We adopted a pragmatic stance, applying inductive and deductive analysis methods.

**Results:**

Participants reported that the FDI stimulated transfer in three ways: 1) Autonomy in creating personal learning objectives and learning processes increased motivation to transfer, 2) Support from peers, supervisors, and students encouraged the adoption of new teaching strategies, 3) Integration of on-the-job experiences and off-the-job meetings fostered a continuous learning cycle of experiencing, reflecting, understanding, and applying.

**Discussion:**

Combining Self-Directed Learning with on-the-job and off-the-job learning within FDIs shows promise in stimulating the transfer of active learning strategies. This approach enables participants to progressively integrate such strategies into their teaching practices. While our findings provide valuable insights for FDI design, further research is needed to evaluate the relative effectiveness of this approach.

## Introduction

Faculty Development Initiatives (FDIs) in medical education typically emphasize the importance of active learning, recognizing its crucial role in stimulating student development [1,2,3,4,5,6]. However, active learning can be difficult for teachers to implement [7,8]. It requires students to meaningfully engage with study materials through (collaborative) activities (such as think-pair-share, or group discussions), with teachers being facilitators of learning processes rather than traditional transmitters of knowledge [2,3,7,9]. This shift requires teachers to develop and use new teaching strategies. Although FDIs are generally effective in enhancing medical teachers’ proficiency in active learning strategies, participants in such initiatives often struggle to apply these strategies in their teaching practice [10,11,12]. Previous research has identified factors that can support teachers’ application of lessons learned [13,14]. However, it remains unclear how to combine these factors in the design of FDIs. The present study addresses this gap to ensure that FDIs can support the implementation of active learning in medical education.

The process by which participants apply lessons from FDIs in their teaching practice is called ‘transfer’. Transfer has been defined as ‘the effective (generalisation) and continuing (maintenance) application in the job environment of the skills, knowledge and conceptions gained in a staff development context’ [13]. Successful transfer has been shown to be associated with a range of positive outcomes, such as enhancing medical faculty well-being, professional development, behaviors, organizational change, student learning, and even patient outcomes [10,11,12]. However, transfer is a complex process influenced by individual, organizational, and pedagogical factors [14,15]. Individual factors include participants’ self-efficacy and their ability to identify learning needs and set relevant goals, affecting motivational barriers to transfer [14,16]. Organizational factors include the constraints FDIs face, such as limited time, insufficient organizational support, and financial restrictions, all of which can hinder the integration of strategies to enhance transfer into their design [10,11,15,17]. Lastly, pedagogical factors include the formal learning environments in which FDIs are often conducted (away from the workplace), creating a gap between the learning and application settings. This gap reduces the likelihood that participants remember and use what they learned [12,13,14,18].

Although several conceptual frameworks to enhance transfer exist, these do not provide a simple solution to this complex challenge. Such frameworks describe variables that previously have been identified to positively impact transfer, but do not prescribe what to do [13,14,15]. To be effective, FDIs should be intentionally designed to address the individual, organizational, and pedagogical barriers that hinder transfer. Among the previously identified variables that have a positive effect, two approaches may be particularly suited to overcome motivational barriers and bridge the gap between learning and application settings. These approaches are Self-Directed Learning, and combining off-the-job learning with on-the-job learning [10,12,13,14,17].

Self-Directed Learning has been defined by a major proponent, Malcolm Knowles, as “a process in which individuals take the initiative, with or without the help of others, in diagnosing their learning needs, formulating learning goals, identifying human and material resources for learning, choosing and implementing appropriate learning strategies, and evaluating learning outcomes” [19]. Self-Directed Learning encourages learners to reflect on what they want to learn and how to achieve that. This process prompts learners to devise a plan that aligns with their job context and to proactively identify solutions to barriers [16]. Off-the-job learning is when FDIs take place away from the workplace and includes structured training, workshops, and other educational courses not directly tied to employee’s daily work tasks. On-the-job learning refers to the learning that occurs when employees engage in their work and includes peer and supervisory support, coaching, organizational learning climates, and other work-related factors [13,14]. While off-the-job learning can help to minimize distractions, be facilitated by expert faculty developers, and take place in controlled locations, on-the-job learning allows FDI participants to acquire experience in the real world [20]. Combining these two concepts in a single approach may stimulate transfer by overcoming the gap between learning and application settings.

Both approaches have demonstrated a positive impact on transfer, but their limitations when used in isolation are also well recognized. For example, Self-Directed Learning may lack structure and the feeling that participants ‘need to do it alone’ [21,22]. Off-the-job learning, as described above, can create a gap between learning and application settings [13,14,18]. On-the-job learning may place high cognitive demands on participants, as learning must occur simultaneously with their regular educational and professional responsibilities [23]. Therefore, combining these approaches potentially allows for a more comprehensive learning process in which the acquisition and application of knowledge and skills are integrated, thus possibly stimulating transfer.

### Aim and research question

The aim of this study was to design and evaluate an FDI focused on stimulating the transfer of competencies related to facilitating small-group active learning methods, and enabling teachers to apply the lessons learned in their own teaching practices. Our main research question for this study was: How does a Faculty Development Initiative that combines Self-Directed Learning with off-the-job and on-the-job learning stimulate the transfer of medical teachers’ competencies in facilitating small-group active learning to their teaching practice?

## Methods

### Research design and procedure

#### Design-based research

Design-Based Research (DBR) aligned well with our aim. DBR aims to systematically design and implement educational interventions, while simultaneously advancing theoretical understanding [24,25,26,27,28]. DBR studies can be characterized by: 1) the use of an iterative process of design, evaluation, and redesign; 2) being conducted in authentic real-life learning settings; 3) its dual aim of solving educational challenges and advancing theory; 4) mixed-method evaluation practices; 5) a collaborative approach of designers, researchers, and practitioners with different expertise [26,29,30].

We adopted a pragmatic stance to data collection and analysis, recognizing that knowledge and action are closely connected in DBR [31,32]. Moreover, the pragmatic paradigm matched well with a mixed-methods research design [33].

#### Development of a prototype FDI

As DBR aims to find sustainable solutions to educational challenges, we developed a prototype that was feasible, sound, locally viable, easy to institutionalize, potentially effective, and positively impacted teacher competencies [27]. Therefore, as a first step, we included faculty developers, course coordinators, educational researchers, study program directors, teachers, and students as stakeholders in this project [34].

The second step was to decide on design principles. In DBR, design principles bridge the gap between theory and practice by offering practical guidelines for the design of a prototype [24,27,34]. In alignment with our research question and aim, our most important overarching design principles were Self-Directed Learning, and the combination of off-the-job learning and on-the-job learning.

The third step was to iteratively refine the prototype design with all stakeholders [27,34]. Throughout, we organized a series of feedback and design sessions to discuss perceptions, experiences, impact, and potential changes. This approach enhanced the relevance and applicability of the prototype while fostering a sense of ownership and commitment among the stakeholders.

Finally, we ran two iterations to evaluate and redesign the prototype, after which we felt that we had optimized it and acquired an understanding of the transfer process and the impact of the FDI. The first iteration started in August 2022 with fifteen participants, and the second in February 2023 with nineteen participants. Based on the feedback and design sessions, and data from the participants, key adjustments made to stimulate transfer included the following: 1) Refining the observation form used during training to better support targeted feedback, 2) Integrating reflection on experiences related to participants’ Personal Learning Objectives with more experienced teachers during the tutor meetings, 3) Refining the Course Day lesson plan to allow more time to formulate Personal Learning Objectives and corresponding plans of actions, 4) Progressively integrating the various components of the FDI design, ensuring that each component built upon the previous one, to create a coherent learning experience. Table 1 shows the final prototype.

**Table 1:** Overview of final prototype design.


COURSE DAY *(off-the-job)*	OBSERVATION *(on-the-job)*	GUIDED PEER COACHING *(off-the-job)*	TUTOR MEETINGS *(both on-the-job and off the-job)*	INDIVIDUALCOACHING *(on-the-job)*	LEARNING WHEN TEACHING *(on-the-job)*

This first meeting was held before the start of the semester, led by supervisor and faculty developer.	After the start of the semester, participants were paired to observe each other in vivo, give each other feedback, and discuss other topics related to their PLO and other teaching experiences. *(SDL 4. OOJ 1)*	Participants participated in guided peer coaching to discuss their PLO, workplace experiences, and other questions *(SDL 4 and 5. OOJ 1, 2, and 3)*	Monthly meeting led by supervisor. During the meeting, all course teachers (i.e., not only the participants) collaboratively reflected on their experiences, prepared for the upcoming classes, and discussed PLO-related questions of the participants. *(SDL 4 and 5. OOJ 1, 2, 3)*	Participants could receive coaching from the supervisor on issues related to active learning as they arose during the semester. *(OOJ 4)*	During the meetings, participants were encouraged to formulate intentions and strategies, try and learn from different approaches to stimulate the active learning processes of their students. *(OOJ 3)*

Preparation for this day included reading course materials, watching knowledge clips, and reading an article on facilitating active learning, including a self-assessment tool on essential facilitator skills. *(SDL 1)*					

During the day, participants would gain the competencies needed to facilitate an active learning process by discussing information, participating in various learning activities, and reflecting on their personal qualities and areas of improvement. *(SDL 1. OOJ 2 and 3)*					

Topics of this course day included ‘creating a safe learning environment’, ‘asking questions’, ‘feedback’, and ‘facilitation skills’.					

At the end of the day, participants would formulate a Personal Learning Objective (PLO), discuss it with peers and facilitators, and develop a plan of approach including an evaluation plan. *(SDL 2, 3, 4 and 5)*					


*Note*. Explanation of information between brackets. SDL = Self-Directed Learning *(1 = diagnosing learning needs, 2 = formulating learning objectives, identifying human and material resources, choosing and implementing learning strategies, evaluating learning outcomes)*. OOJ = On- and Off the Job Learning *(1 = peer support, 2 = supervisory support, 3 = transfer climate, 4 = coaching)*.

### Participants and Setting

The 34 participants in this study had recently started as teachers of a tutoring course in years 1 and 2 of the Bachelor’s phase of medical training at the Medical Faculty of the Vrije Universiteit Amsterdam. They represented a mix of various medical and research backgrounds, with some having limited teaching experience and others none. Participants did this teaching task alongside their main appointment as clinicians or researchers.

The tutoring course was a small-group active learning course. Teachers in this course facilitated active learning processes for their study groups of up to twelve students, meeting twice weekly for the duration of a semester. The course employed a collaborative case-based learning approach using patient cases and assignments [5,35]. In the first weekly meeting, students collaboratively brainstormed the cases and assignments. Then, between meetings, students would meet in subgroups to complete the assignments. In the second meeting, the subgroups presented their findings and discussed them with the full group. Meetings were student-led and learning the content was their responsibility, allowing teachers to focus on student participation and group dynamics.

### Instruments

To comprehensively understand the effects of the prototype, we used a concurrent mixed-methods approach [33]. We chose to collect self-reported data, as we were interested in participants’ perceptions regarding their learning and transfer processes. Quantitative data were collected through a survey we developed for this study (see Table 2), using conceptual definitions for item construction. The survey was filled out following the Course Day (before the start of the semester) and guided peer coaching (a few weeks into the semester) to evaluate: 1) Self-Directed Learning (6 items); 2) active learning competencies (5 items); and 3) motivation to transfer (2 items). We included ‘motivation to transfer’ in the survey to assess intention and readiness to apply what was learned), due to limited actual transfer opportunities. Motivation to transfer has been shown to be an important precursor for actual transfer [14].

**Table 2:** Combined participant survey data (N = 34 over two iterations).


	ITERATION 1 (N = 15)			ITERATION 2 (N = 19)		
	
	MEAN	SD	MEDIAN	MEAN	SD	MEDIAN

Course day						

*Self-Directed Learning*						

I have a better understanding of my strengths and areas for development in teaching a small-group active learning class	3.87	0.92	4	3.74	0.99	4

I think setting a personal learning objective is a good way to stimulate my development	4.47	0.64	5	4.47	0.61	5

I have a plan for achieving my personal learning objective	4.27	0.88	4	4.26	0.81	4

I know how to deal with obstacles should they arise	4.40	0.51	4	4.68	0.58	5

*Motivation to transfer*						

I feel motivated to use the knowledge and skills I learned during the course day	4.47	0.64	5	4.58	0.51	5

I know how to apply the knowledge and skills I learned during the course day	4.40	0.63	4	4.32	0.67	4

*Competencies in facilitating active learning*						

I am aware of challenges related to teaching a small-group active learning class	4.33	0.72	4	4.63	0.50	5

I know how to deal with challenges related to teaching a small-group active learning class	4.20	0.56	4	4.32	0.48	4

I am aware of best practices related to teaching a small-group active learning class	4.27	0.59	4	4.37	0.76	4

I know how to implement best practices related to teaching a small-group active learning class	4.20	0.68	4	4.26	0.81	4

I feel competent in facilitating the active learning processes of students (before and after course day)	3.27–4.40	0.70–0.51	3–4	2.79–4.00	0.71–0.33	3–4

Guided Peer coaching	**(N = 8)**			**(N = 12)**		

*Self-directed Learning*						

I intend to keep on working on my personal learning objective	4.75	0.46	5	4.83	0.39	5

I know how I will keep working on my personal learning objective	4.50	0.53	4.5	4.67	0.49	5

*Motivation to transfer*						

I feel motivated to use the knowledge and skills I learned during peer coaching	4.50	0.53	4.5	4.83	0.39	5

I know how to apply the knowledge and skills I learned during peer coaching	4.63	0.52	5	4.50	0.52	4.5

*Competencies in facilitating active learning*						

I am aware of challenges related to teaching a small-group active learning class	4.75	0.46	5	4.83	0.39	5

I know how to deal with challenges related to teaching a small-group active learning class	4.25	0.46	4	4.50	0.52	4.5

I am aware of best practices related to teaching a small-group active learning class	4.38	0.52	4	4.33	0.89	4.5

I know how to implement best practices related to teaching a small-group active learning class	4.38	0.52	4	4.25	0.87	4

I feel competent in facilitating the active learning processes of students (before and after guided peer coaching)	3.88–4.13	0.64–0.35	4–4	3.58–4.25	0.51–0.62	4–4


*Note*. Answers could be given on a scale from 1 to 5. 1 = completely disagree and 5 = completely agree. Differences in N between course day and guided peer coaching were mainly due to scheduling conflicts.

Qualitative data were collected through semi-structured individual interviews at the end of the semester by JWG or RG to explore how the design principles stimulated transfer and the FDI’s effectiveness in developing active learning competencies. The interviews took approximately 45 minutes, and Appendix 1 provides the interview guide.

Data collection was consistent across iterations. This means that there were six moments of data collection in total: two quantitative (Table 2) and one qualitative per iteration.

### Data Analyses

Quantitative data were analyzed using descriptive statistics in IBM SPSS statistics (version 28). For analysis of the qualitative data, we used Atlas.ti (version 22). All interviews were audio-recorded and transcribed. Authors JWG and RG conducted the coding of data, frequently discussing their progress with each other and the broader author team to identify and question patterns, reflections, and other insights. The first iteration’s qualitative data were analyzed using Inductive Thematic Analysis [36,37], with design principles applied as sensitizing concepts. Following recommended procedures, the codes and discussions were used to identify and define themes [36]. For the second iteration, the resulting themes formed a coding framework for qualitative data analyses. We then used Directed Content Analysis to corroborate the first iteration’s findings while remaining open to new information [38]. Although this analysis largely confirmed the original themes, some new insights emerged, particularly related to the value of alternating on-the-job and off-the-job learning opportunities, which were integrated into the theme descriptions.

Following our pragmatic stance, all data were combined to answer the research question. Insights from each data source were assessed, compared, and evaluated for their contribution to our understanding. The author team repeatedly engaged in this process through group discussions, each time reflecting how new insights related to previous ones, progressively expanding and deepening our understanding of the learning and transfer processes in this FDI.

### Reflexivity

The author team collectively possessed experience and training in active learning methods, faculty development, educational design, educational research, and Design-Based Research. Two authors had practical experience with the tutoring course: one was an experienced teacher, while another was a medical student who had done the tutoring course. We valued the different perspectives that the researchers brought to the study while remaining aware that this may have influenced the design and evaluation of the prototype. For instance, during the analyses of qualitative data, authors had varied perspectives on including students as a source of support, indicating how it could also reduce students’ willingness to participate. Ultimately, we agreed to include student support as part of the second theme (described below) because, according to participants, it supported their learning and transfer. This outcome shows how such discussions helped our interpretation of the data. Therefore, we remained reflexive throughout the research process and acknowledged our subjectivity.

### Ethical aspects

Ethical approval was obtained from the Ethical Review Board of the Netherlands Association for Medical Education (NVMO-ERB dossier number 2022.5.4).

## Results

The evaluation of the two iterations is described together here, to present the findings coherently and concisely.

Table 2 presents the combined participant survey data from both iterations of the FDI. Answers could be given on a scale from 1 (completely disagree) to 5 (completely agree). Across all categories (Self-Directed Learning, motivation to transfer, and competencies in facilitating active learning), the median scores for most items were 4 and 5, indicating a high level of agreement among participants. The notable exception was the before and after feeling of competency in facilitating active learning, answered at the end of the Course Day in both iterations.

The interview data (N = 28) uncovered how, according to participants, Self-Directed Learning and the integration of on-the-job and off-the-job learning stimulated transfer. Three themes were identified: 1) Autonomy in creating personal learning objectives and learning processes enhanced motivation to transfer, 2) Support from peers, supervisors, and students stimulated the adoption of new teaching strategies, and 3) Integration of on-the-job experiences and off-the-job meetings stimulated a continuous learning cycle of experiencing, reflecting, understanding, and applying.

**1) Autonomy in creating personal learning objectives and learning processes enhanced motivation to transfer**

Participants who had volunteered to be teachers in the course reported feeling motivated by the autonomy to formulate a Personal Learning Objective and a plan of approach at the end of the initial Course Day, enabling them to develop skills that were personally relevant and instilling a commitment to grow.

*[Without the FDI], I wouldn’t have set a learning objective. I believe that on the course day, during the formulation [of the Personal Learning Objective], there was an emphasis on taking action and reflecting on how it’s going. This emphasis on action and reflection was also reiterated in the following meetings. So, in that sense, it encouraged a proactive approach to teaching and reflecting on how you teach. (iteration 2, participant 9)*

Participants for whom teaching was mandatory (e.g., teaching as part of their research job or specialist training), initially reported feeling less commitment to their growth.

*I am placed in this role, but I also don’t want to shortchange the students. […] They are the doctors of the future, and I do want them to be motivated by their studies and learn a lot. So, in itself, I do consider it important to take my role seriously and do my best. (Iteration 2, participant 14)*

However, the autonomy given also allowed this second group to formulate objectives and plans of approach that suited them and their work context, enhancing focus and motivation. They could spend their time on activities that were relevant to them. During their teaching, all participants found ways to work on their Personal Learning Objectives.

*I wanted to create a safe group. I had set that Objective because I thought that was really the most important thing. That they feel safe with each other, that they can say anything. That is why I think I was much more personal, and I also reflected on how the group was doing in this regard. How is it right now? Should we do something about it? And I also very proactively asked them, for example, during a formative assessment meeting: “Is this a good group for you?” And if not, what would they want differently or where and when it would be a good group for them. (iteration 1, participant 9)*

During follow-up meetings, participants shared insights related to their Personal Learning Objectives but also questions and obstacles. The conversations that occurred at those meetings further supported participants’ motivation, as they felt inspired and supported by other participants. Participants who missed these meetings did not experience the same outcomes.

**2) Support from peers, supervisors, and students stimulated the adoption of new teaching strategies**

Facilitating small-group active learning was reported to be a challenging skill, even for participants who had received prior training. Applying FDI competencies in their teaching practice involved managing feelings of uncertainty and potential failure. For this reason, participants required support. They reported seeking support through individual coaching from their supervisor and asking questions of colleagues. During FDI meetings, they sought the support of fellow participants and supervisors.

*I found the peer coaching very useful, where we discussed cases. And that is exactly what you need. You can’t prepare yourself that well for teaching because things will go a certain way. And during the peer coaching, you can discuss things very concretely. For instance, the case we had about certain student behaviors. And how you can handle that. And I think that was the most useful for me personally. To see what other teachers are running into and that it is ‘not strange’ [what I am running into and what I have difficulty with]. (iteration 1, participant 2)*

Finally, participants who had opened up to their students about being a first-time teacher and communicated their intentions and openness to feedback and learning, reported receiving valuable information about how they facilitated the active learning process. They reported that they felt supported by their students through constructive comments they received about instances that could have been handled better and compliments about things that were going well. This influenced their sense of learning and self-efficacy.

**3) Integration of on-the-job experiences and off-the-job meetings stimulated a continuous learning cycle of experiencing, reflecting, understanding, and applying**

According to the participants, there was a synergy between on-the-job experiences and regular off-the-job meetings throughout the semester, as each inspired the other. The on-the-job experiences were reflected upon during the meetings, so that on the one hand learning from those experiences was stimulated, and on the other hand guidelines were created for future teaching practice. Applying those guidelines led to new experiences, in effect creating a dynamic learning cycle where theory and practice were interwoven.

*The training was for first-time tutors. It [the FDI] should guide someone on how to start. Ensure they have enough tools so that they can at least start their study groups and guide the students. […] I think that the initial course day ensured that we could actually get started. And between the meetings, it [the FDI] ensured that I remained sharp on where I am working on now and how I can proceed. You need to continue developing your skills, because you can’t just say: you’ve had training, here is your tutor certificate, you can now facilitate study groups perfectly from now on. It does not work like that, so there needs to be something. I think there was a good balance. You have a few study group meetings, and then you get together again [with peers and supervisor] to discuss how this period went. (iteration 2, participant 15)*

The participants appreciated this approach, as it enriched their learning experiences and promoted a culture of continuous improvement and adaptation in their teaching practices.

*I can’t imagine that you can teach someone a skill after just one training session. […] I think you need a combination of actually doing the teaching itself and the guidance provided [by the FDI]. That helps, but not one without the other. […] So, do, reflect, prepare, do, reflect, do, reflect, and so on. (iteration 2, participant 9)*

## Discussion

In this study, we explored how a faculty development initiative (FDI) that combines Self-Directed Learning with off-the-job and on-the-job learning stimulates the transfer of medical teachers’ competencies in facilitating small-group active learning. Firstly, the results indicate that the FDI indeed supported teachers in developing their active learning facilitation skills and applying them in their teaching practices. Secondly, the FDI made an impact through stimulating teachers to develop personally relevant skills, while providing support and a structured approach to progressively integrate these skills into their teaching practices. Alternating workplace learning and formal training sessions was crucial for this purpose. Facilitating active learning was confirmed to be a complex skill, which benefited from sustained FDI support.

This study contributes to the literature on transfer by demonstrating that Self-Directed Learning can stimulate transfer by enhancing the autonomy and motivation to transfer of FDI participants. FDIs can use these insights by allowing participants to choose which skills they want to develop and devise an implementation plan that suits their needs and contexts. Typically, FDIs are designed with predetermined objectives, to ensure alignment with institutional goals, course requirements, or specific teaching competencies. While such objectives provide structure and clarity, they can limit transfer if participants feel the objectives are not relevant for them [13,14,16,21].

Previous transfer studies had already provided evidence that learning on-the-job, off-the-job, and the combination can stimulate transfer [13]. This study highlights how the combination specifically supports transfer: by creating a continuous learning cycle of experiencing, reflecting, understanding, and applying. This finding advances understanding of transfer processes and provides an actionable approach to FDI design. Many FDIs are designed as one-time events or short courses [12,18]. Such designs were already shown to have limited impact and more extensive initiatives may be able to better integrate on-the-job and off-the-job learning opportunities to strengthen transfer strategies.

A final contribution to the literature is that the combination of Self-Directed Learning, off-the-job, and on-the-job learning can alleviate some of the criticism of these concepts in isolation. Self-Directed Learning requires that learners be responsible for their development, which can lead learners (and teachers) to think that they need to do it alone [19,21]. In situations without proper guidance, Self-Directed Learning can be perceived by learners as a synonym for lack of support [22]. On-the-job learning can provide peer and supervisor support through sharing on-the-job experiences, feedback, and coaching. However, on-the-job learning has been criticized for placing a high cognitive load on learners, especially on novices, resulting in less optimal learning [23]. In situations with high cognitive load, the focus that Self-Directed Learning can bring might enhance learning by reducing the experience load. Off-the-job learning takes place away from the workplace, in a focused, structured, and facilitated setting, thereby again reducing load. However, since off-the-job learning has been shown to create a gap between learning and application settings [13,14], it can benefit from the real-world experiences that on-the-job learning can deliver. Thus, the combination of the three concepts can stimulate transfer by providing a supportive, responsive, focused, and authentic learning experience.

Although the design of this FDI requires organizational investments, due to the multiple components of the training, use of trained faculty developers, and time investment by the participants, it may still provide a solution to previously identified constraints in faculty development. Usually, participants and organizations want training that requires little time, finances, and organizational support as each of these resources is limited [10,11,13,14,17]. By facilitating participants to ‘learn while teaching’, as well as leveraging peer and supervisory support, the design of this FDI reduces the need for sessions away from work and minimizes reliance on trained faculty developers. However, we acknowledge that this approach does not completely eliminate time and other types of investments and additional research would be needed to further justify it.

### Strengths and limitations

The DBR approach enabled us to develop, implement, and evaluate the impact of an FDI in a natural setting. We conducted the study with a diverse team, guided by theory, and employed an iterative and mixed-methods approach. The combination of immediate post-training quantitative data (which seems to show overconfidence in participants’ learning and transfer given the high scores) with end-of-semester qualitative data (which shows that implementing the lessons learned and transferring acquired skills was more difficult than participants anticipated) contributed to both addressing an educational challenge and transfer. Although the use of self-reported data provided an in-depth understanding of participants’ perceptions of their learning and transfer, these perceptions were not corroborated with additional sources of data. Self-report in transfer literature has been shown to show stronger associations with influencing variables than other sources of data [14]. Furthermore, the FDI was designed and implemented in a specific context, requiring reflection and adaptation before transferring it to other institutions.

### Future research

While our findings provide valuable insights for FDI design, further research is needed to evaluate the relative effectiveness of this approach. A (quasi-) experiment may be best suited for this purpose, as it allows the comparison of groups. Observational and student data can be used to assess teachers’ actual transfer, performance, and impact on student development. These suggestions may lead to a more comprehensive understanding of how new medical teachers can learn to facilitate active learning.

## Conclusion

The faculty development initiative we designed combined a Self-Directed Learning approach with on-the-job and off-the-job learning opportunities, demonstrating it to be feasible and responsive to the needs of teachers. Consequently, participants felt stimulated to apply active learning strategies to their teaching practices.

## Appendix 1. Participant interview guide

**Table:**

STRUCTURE OF THE INTERVIEW	EXAMPLE QUESTIONS

1. Opening questions *(Goals: getting comfortable; activating relevant experiences and information)*	- How was your first semester as a teacher? - What were some things that you liked/did not like / that surprised you (either in a good or bad way)?

2. General FDI questions *(Goal: reflecting on whether and how the FDI supported participants)*	- Which parts of the training did you participate in? How did (or did not) each part contribute to your competencies in guiding the active learning processes of your students? - Overall, how well do you think you were prepared and guided in learning to facilitate the active learning process of students? - To what extent have you used the knowledge and skills you learned in the training in your own study groups? What impact do you think this has had on the study group?

3. Design Principle questions *(Goal: Reflecting on whether and how the design principles supported participants)*	- One aim of the training was to stimulate you to take control of your own learning process and develop yourself on something relevant to you throughout the semester. How did this work for you? What did you think about having a Personal Learning Objective? How do you think it influenced your development as a teacher this semester? - Another aim of the training was to stimulate you to learn from your workplace experiences. For that reason, we had the regular meetings throughout the semester. You could gain experience in your teaching tasks and then meet with your peers in various meetings to discuss them. How did this work for you? How do you think it influenced your development as a teacher this semester?

4. Closing questions	- Did you seek advice for your teaching task outside the training? - How do you think the FDI could be optimized? What could be kept as it is and what could be changed, added, or deleted?
